# Dietary β-Carotene Rescues Vitamin A Deficiency and Inhibits Atherogenesis in Apolipoprotein E-Deficient Mice

**DOI:** 10.3390/nu12061625

**Published:** 2020-06-01

**Authors:** Ayelet Harari, Nir Melnikov, Michal Kandel Kfir, Yehuda Kamari, Lidor Mahler, Ami Ben-Amotz, Dror Harats, Hofit Cohen, Aviv Shaish

**Affiliations:** 1 The Bert W. Strassburger Lipid Center, Sheba Medical Center, Tel-Hashomer 5262000, Israel; nirmelnikoc@gmail.com (N.M.); michal.kandelkfir@sheba.gov.il (M.K.K.); yehuda.kamari@sheba.gov.il (Y.K.); imulo43@googlemail.com (L.M.); dror.harats@sheba.gov.il (D.H.); hofit.cohen@sheba.gov.il (H.C.); aviv.shaish@sheba.gov.il (A.S.); 2 The Sackler School of Medicine, Tel-Aviv University, Tel-Aviv 6997801, Israel; 3 N.B.T., Nature Beta Technologies LTD, Eilat 8851100, Israel; amiba@sheba.gov.il; 4 The Department of Life Sciences, MP, Achva Academic College, Shikmim 7980400, Israel

**Keywords:** β-carotene, vitamin A, atherogenesis, liver, adipose tissue

## Abstract

Vitamin A deficiency (VAD) is a major health problem, especially in developing countries. In this study, we investigated the effect of VAD from weaning to adulthood in apoE^−/−^ mice. Three-week-old male mice were allocated into four diet groups: I. VAD II. VAD+vitamin A (VA), 1500 IU retinyl-palmitate; III. VAD+β-carotene (BC), 6 g/kg feed, containing 50% all-trans and 50% 9-cis BC. IV. VAD with BC and VA (BC+VA). After 13 weeks, we assessed the size of atherosclerotic plaques and measured VA in tissues and BC in plasma and tissues. VAD resulted in diminished hepatic VA levels and undetectable brain VA levels compared to the other groups. BC completely replenished VA levels in the liver, and BC+VA led to a two-fold elevation of hepatic VA accumulation. In adipose tissue, mice fed BC+VA accumulated only 13% BC compared to mice fed BC alone. Atherosclerotic lesion area of BC group was 73% lower compared to VAD group (*p* < 0.05). These results suggest that BC can be a sole source for VA and inhibits atherogenesis.

## 1. Introduction

According to the World Health Organization (WHO), millions of children in developing countries suffer from vitamin A deficiency (VAD) [1,2]. Vitamin A (VA), mostly defined as all-trans retinol [3], has many roles in the human body, including growth and reproduction, vision, epithelial differentiation, lipid metabolism, and immune function [4]. 

Barker et al. suggested that poor nutrition in early life and low birth weight are associated with an increased risk of ischemic heart disease in adulthood [5]. The association of VAD with cardiovascular risk factors has been studied in children, adolescents, and adults. Plasma vitamin A insufficiency was associated with obesity, lower levels of high density lipoprotein (HDL), hypertriglyceridemia and metabolic syndrome [6,7,8]. In children in low-income regions, VAD is a result of inadequate VA intake or low dietary fat, which leads to insufficient absorption of VA. Other risk factors for VAD in the general population, include chronic liver disease, inflammatory bowel disease, recurrent pancreatitis, excessive alcohol consumption, iron deficiency and bariatric surgery [9,10,11,12,13,14]. The WHO recommends VA supplementation for children in VAD areas [15]. On the other hand, excess VA intake may be teratogenic and lead to congenital birth defects [16,17], increased risk for osteoporosis and bone fractures [18]. While in most western populations, meat and dairy products serve as a main dietary source of VA, in the growing vegan population, pro-VA carotenoids from plants are the only source of VA. 

It has been suggested that increased consumption of pro-VA β-carotene (BC) enriched foods can increase body stores of VA [19,20]. The alga *Dunaliella bardawil* is a natural source of BC, containing mainly all-trans and 9-cis BC isomers. Although both isomers are present in fruits and vegetables, synthetic BC comprises only the all-trans isomer [21]. In this respect, it is important to note that human trials that failed to demonstrate a beneficial effect of BC supplementation on cancer or cardiovascular disease used the synthetic, all-trans isomer [22,23,24]. We showed that dietary enrichment of mice with synthetic all-trans BC accelerated atherogenesis, while a diet containing algal powder with 9-cis and all-trans BC or isolated 9-cis BC inhibited atherosclerosis development [25,26,27]. These findings highlight the importance of the 9-cis BC in the diet. 

In a previous study, we demonstrated that BC from the alga *Dunaliella bardawil* compensated for VAD and repressed VAD induced-atherogenesis in adult mice. Moreover, the addition of a natural BC to a VAD diet increased hepatic levels of BC more than the addition of BC to a VA-containing diet [28]. These results are in accord with a previous study in chicks which showed a consistent decrease in serum, liver and skin carotenoid content with increasing dose of dietary VA [29]. Another study performed in cattle showed that high dietary VA decreased plasma and liver carotenoid concentrations [30]. In humans, subjects with greater intake of VA absorbed less BC and converted it to VA at a lower rate than individuals with lower intake of VA [31]. Nevertheless, data on the effect of dietary VA or its plasma and tissue levels on BC bio-distribution are limited. This motivated us to investigate, at the present study, whether VA affects the bio-distribution of BC and its stereoisomers in animal tissues.

9-cis BC is a source for 9-cis retinoic acid (RA) that serves as a ligand for the nuclear receptor retinoid X receptor (RXR) [32]. RXR plays a role in numerous atherogenesis-related pathways, including lipid metabolism, inflammation and reverse cholesterol transport [33,34,35,36], a process which may inhibit atherosclerosis by transporting cholesterol from the blood vessels to the liver. We showed that feed fortification with natural BC, containing 9-cis and all-trans isomers, accelerated cholesterol efflux from macrophages to HDL [37]. The effect of dietary VAD on atherogenic risk factors has also been investigated. In rat models, VAD diet decreased total plasma cholesterol, HDL cholesterol and triglycerides levels [38,39], while in adult apoE^−/−^ mice, VAD increased plasma cholesterol [28]. To the best of our knowledge, there is no data regarding the effect of VAD diet in models for atherosclerosis from adolescence to adulthood. In the current study, we sought to investigate the effect of a VA-containing vs. VAD diet on tissue VA and atherogenesis in apoE^−/−^ mice from weaning to adulthood; to examine whether dietary BC, as a sole source of VA, could compensate for dietary VAD; and to investigate the effect of dietary combination of VA and BC on tissue VA and BC bio-distribution.

## 2. Materials and Methods 

### 2.1. Mice

After weaning from suckling, 39 male, 3-week-old apoE^−/−^ mice (C57BL6 background, Jackson Laboratories) were used. The animals were housed in plastic cages on a 12 h light/12 h dark cycle with free access to feed and water. At the end of the experiment, the mice were killed with isoflurane. The Animal Care and Use committee of Sheba Medical Center, Tel-Hashomer, approved all animal protocols (Helsinki protocol no 682/11).

### 2.2. Diet

We used a VAD diet (17.7% protein, 5% fat; TD86143, Harlan Teklad) and a powder of the alga *Dunaliella bardawil* (Nikken Sohonsha, Japan) as a source for natural carotenoids. The algal powder contains 6% BC (weight/weight), composed of 50% all-trans and 50% 9-cis isomers of BC [21]. VA as retinyl palmitate (Solgar, dry VA 1500 mcg (5000 IU) tablets) was used. To prepare the feed, 0.25 L of hot distilled water was mixed with 14 g of gelatin until the solution was clear. Then, 1 kg of powdered feed and BC powder (80 g/kg feed), VA, or a combination of VA and BC powder were thoroughly mixed with the warm gelatin solution. After solidifying, the feed was divided into tablets and stored at −20 °C. The feed was replaced every other day to minimize oxidation and degradation of its ingredients. During pregnancy, dams were fed a regular chow diet containing 18% protein and 5% fat, containing 4.5 μg vitamin A/g (TD2018, Harlan Teklad). 

### 2.3. Study Design

Mice were allocated into four groups, 10 animals per group, according to their body weight and fed for 13 weeks with the specified diet. I. VAD II. VA: the VAD feed was fortified with 1500 IU VA per kg diet. III. BC: the VAD feed was fortified with 8% BC powder, (weight/weight), containing 6 g BC (50% all-trans and 50% 9-cis BC), IV. BC+VA: the VAD feed was fortified with BC and VA. 

### 2.4. Lipid Analysis

We used a colorimetric enzymatic procedure to measure total plasma cholesterol (Chol, Roche/Hitachi, Roche Diagnostics) and triglycerides (TG) (Triglyceride liquid, Senitinel).

### 2.5. Carotenoid and Retinol Analysis

All tissues were stored at −80 °C and analyzed together. Plasma (~100 μL) and tissue (~150 mg) samples were extracted with 2 mL (3 mL for adipose and brain tissue) of ethanol containing 10 µM butylated hydroxytoluene. After the addition of 2 mL hexane for plasma, liver and renal, 4 mL for brain and adipose tissue, and 1 mL DDW, the samples were mixed and centrifuged for 5 min at 1000 g. The hexane layer of plasma samples was divided to two aliquots (one for VA and one for carotenoid detection). The hexane layers of liver and adipose tissue samples were saponified with 12% KOH in absolute ETOH for 30 min, at 50 °C. After washing twice with 2 mL of saline followed by centrifugation, the hexane layer was dried under a stream of N_2_. Dried samples were suspended in 100 µL tert-butyl methyl ether for carotenoids and 100 µL methanol for VA detection. VA (all-trans retinol) concentrations were determined by reverse phase HPLC on a Vydac C18 column (201TP-54, 250 × 5 mm, 5 μm particle size; Vydac, Hesperia, CA) with methanol/butanol/water 10 mM ammonium acetate as the mobile phase, at a flow rate of 0.8 mL/min. Retinyl acetate was used as an internal standard for retinol detection. Serum carotenoids were determined by reverse phase HPLC on YMC C30 column (CT995031546QT, 150 × 4.6, 3 μm particle size; YMC Inc., Allentown, PA, USA) with a gradient, as described [40]. Carotenoids were detected by monitoring absorbance and by comparison with the retention times of authentic standards. Absorbance was detected at wavelength of 200 to 700 nm in order to identify carotenoids with absorption in the UV range and the visible range of the spectrum. 

### 2.6. Assessment of Atherosclerosis in the Aortic Sinus

Atherosclerotic fatty streak lesions were quantified by calculating the lesion areas in the aortic sinus [41]. 

### 2.7. Analysis of Gene Expression by Real-Time PCR

A Nucleospin RNA II kit (Macherey-Nagel) was used for RNA extraction. A high capacity cDNA synthesis kit (Applied Biosystems) was used to perform cDNA synthesis. Quantitative real-time PCR was performed with a 7900HT PCR machine (Applied Biosystems), FastStart Universal Probe Master ROX (Roche), and a FAM-labeled TaqMan primer and probe (Ap plied Biosystems and Roche) for Cytochrome P450 Family 26 Subfamily A Member 1 (*Cyp26a1*; 312454, Roche), *Cyp26b1* (316002, Roche), *LDL R* (307884, Roche), Beta-Carotene 15,15’-Monooxygenase 1 (*Bco1*; 316000, Roche), *Bco2* (31690, Roche), Retinaldehyde Dehydrogenase, type I (*Raldh1*; 318333, Roche ), Retinol-Binding Protein 4, (*Rbp4*; 318335, Roche ). We used *Gapdh* as a reference gene (307884, Roche). 

### 2.8. Statistical Analyses

One-way ANOVA was used to compare the treatment effect on atherogenesis and on VA and BC levels with the post-hoc Tukey method used for multiple pairwise comparisons. Repeated-measures ANOVA was applied to compare changes in weight gain between the treatment groups over the study period. Significance was considered as *p* < 0.05. *t*-test was used to test the difference between 9-cis BC percent in BC group compared to BC+A group (*p* < 0.05). Values are means ± SE.

## 3. Results

### 3.1. Body Weight

**Figure 1:**
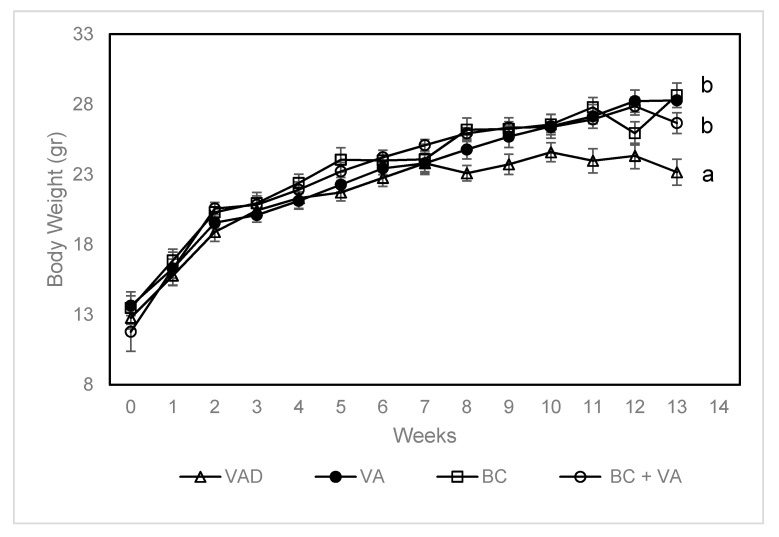
Body weight (gr) throughout the study, in apoE^−/−^ mice fed with VAD diet, VA, BC or BC+VA. Data are means ± SE, n = 8–10 in each group. Mean values (a,b) not sharing a common superscript were significantly different by repeated measures ANOVA and Tukey post-hoc test comparison (*p* < 0.05). VAD = vitamin A deficient, V = vitamin A, BC = β-carotene.

We measured body weight throughout the 13-week experiment. During the first seven weeks, the increment in body weight was similar in all groups. From the 8th week and forward, mice in the VAD group stopped gaining weight, whereas the mice in all the other groups continued to gain weight in a constant rate (Figure 1). Body weight of BC and BC+VA was significantly higher and tended to be higher in the VA (*p* = 0.064) compared to VAD group. 

### 3.2. Tissue VA Levels 

**Figure 2:**
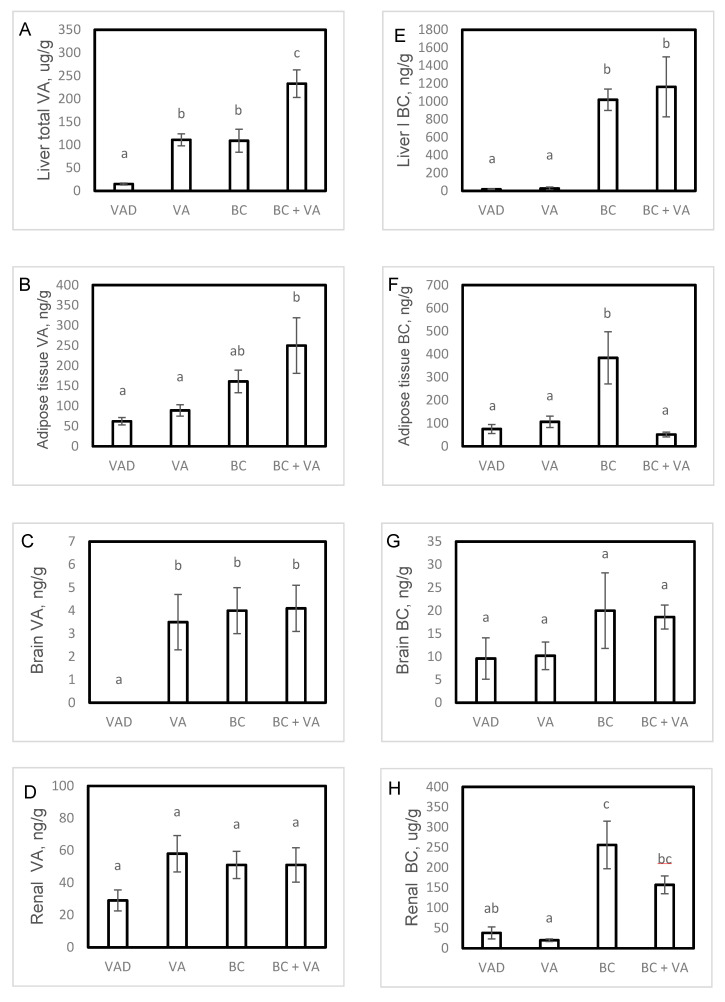

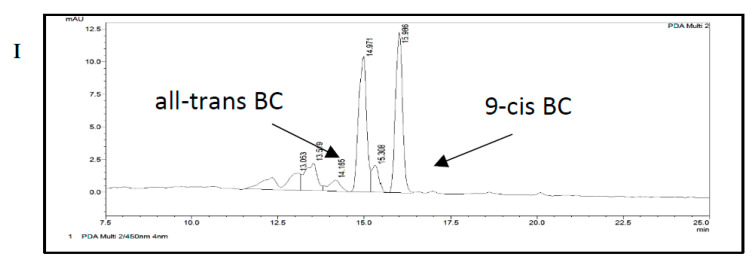
Tissue VA and BC (**A**–**H**) concentrations in apoE^−/−^ mice fed with VAD diet, VA, BC or BC+VA. Data are means ± SE, n = 6–10 in each group. Mean values (a–c) not sharing a common superscript were significantly different by one-way ANOVA and Tukey post-hoc test comparison (*p* < 0.05). Representative HPLC analysis of hepatic carotenoids (spectrum range 200-700 AU) (**I**). VAD = vitamin A deficient, VA = vitamin A, BC= β-carotene.

We measured VA (all-trans retinol) levels in tissues after 13 weeks and found that hepatic VA levels were significantly higher in VA, BC and BC+VA groups, compared to the VAD. Dietary BC, as a sole source of VA, completely restored hepatic VA levels and the combination of VA+BC led to two-fold VA accumulation compared to VA or BC alone (Figure 2A). Epipidymal adipose tissue VA levels were significantly higher in BC+VA group compared to VAD (Figure 2B). In the brain, similar VA levels were detected in VA, BC and BC+VA, while in the VAD group, VA was undetectable (Figure 2C). In the kidneys, no significant differences were found between the groups (Figure 2D).

### 3.3. Tissue BC Levels

Plasma BC levels were significantly higher in the BC group and tended to be higher in the BC+VA group compared to VAD (*p* = 0.089, Supplementary Table S1). In the liver, BC alone or combined with VA resulted in a similar and significant greater accumulation of BC compared to VAD (Figure 1E). In contrast, in epididymal adipose tissue of mice fed BC alone, there was a substantially greater accumulation (7-fold increase) of BC compared to mice fed the combination of BC+VA. Moreover, BC levels in the BC+VA group were similar to the levels in the VAD group (Figure 1F). In the kidneys, BC levels were significantly higher in BC group and tended to be higher in BC+VA group compared to VAD (*p* = 0.071, Figure 2H). Total BC was calculated as the sum of 9-cis and all-trans isomers, and both isomers were detected in all tissues. The percentage of the 9-cis BC isomer was tissue- and treatment-dependent. The relative amount of 9-cis was higher in mice fed BC. However, in adipose tissue and kidneys, 9-cis BC was significantly lower in the combined BC+VA treatment compared to BC alone (Supplementary Table S1). 

### 3.4. Plasma Cholesterol and Atherogenesis 

**Figure 3:**
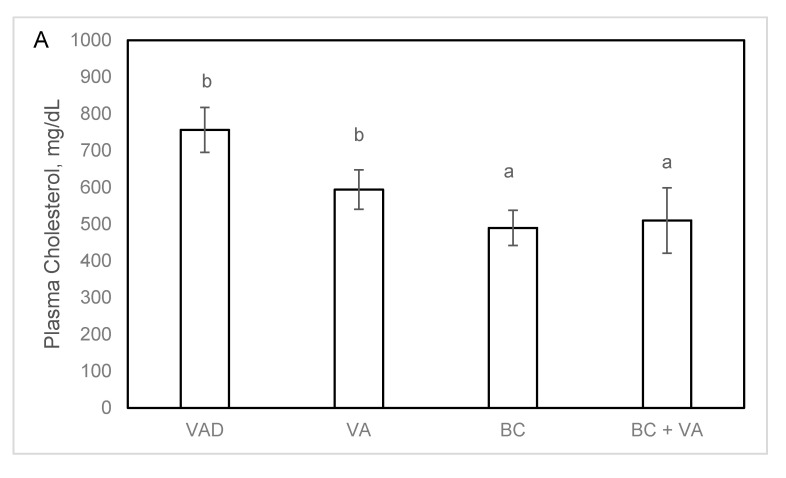

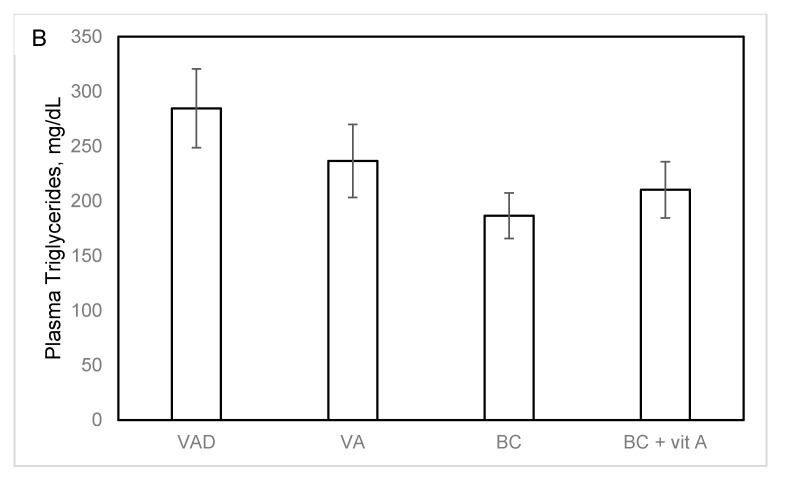
Plasma cholesterol (**A**) and triglycerides (**B**) concentrations in apoE^−/−^ mice fed with VAD diet, VA, BC or BC+VA. Data are means ± SE, n = 8–10 in each group. Mean values (a,b) not sharing a common superscript were significantly different by one-way ANOVA and Tukey post-hoc test comparison (*p* < 0.05). VAD = vitamin A deficient, VA = vitamin A, BC = β-carotene.

**Figure 4:**
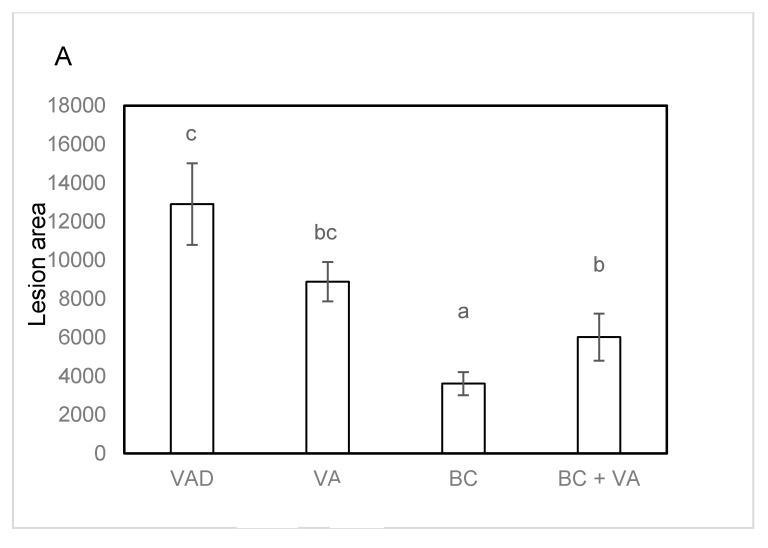

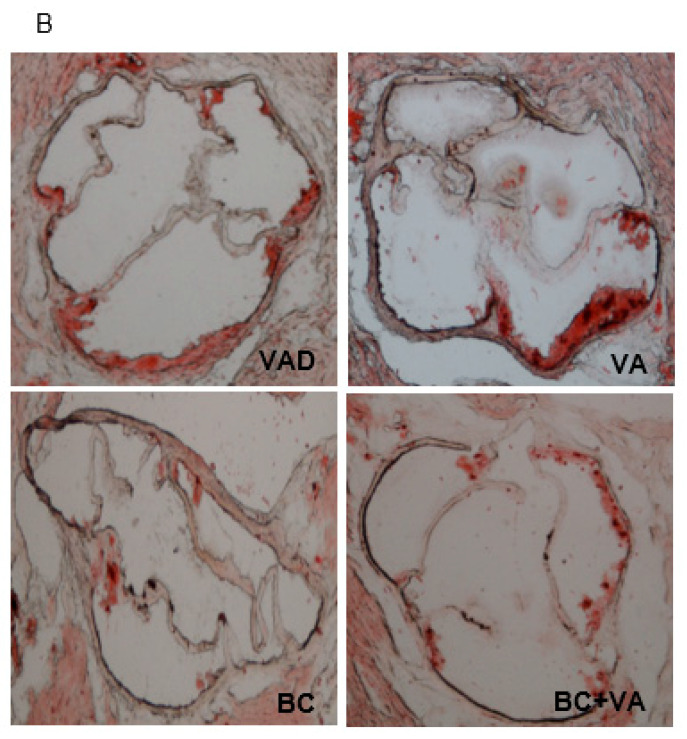
Atherosclerotic lesion area (**A**) and representative photographs of aortic sinus lesion section (magnification ×40), one for each group. Red color indicates the presence of atherosclerotic lesions (**B**) in apoE^−/−^ mice fed with VAD diet, VA, BC or BC+VA (A). Data are means ± SE, n = 6–8 in each group. Mean values within a row (a–c) not sharing a common superscript were significantly different by one-way ANOVA and Tukey post-hoc or Kruskal–Wallis test comparison (*p* < 0.05). VAD = Vitamin A deficient, VA = vitamin A, BC = β-carotene.

BC supplementation significantly reduced plasma cholesterol levels in both BC and BC+VA groups after 13 weeks of treatment compared to VAD group (Figure 3A). Both BC and BC+VA treatments significantly reduced the atherosclerotic lesion area in the aortic sinus compared to VAD (73% and 54%, respectively), while the lesion in the VA group wasn’t significantly lower compared to the VAD group (Figure 4A).

### 3.5. The mRNA Levels of RA Catalyzing Enzymes

**Figure 5:**
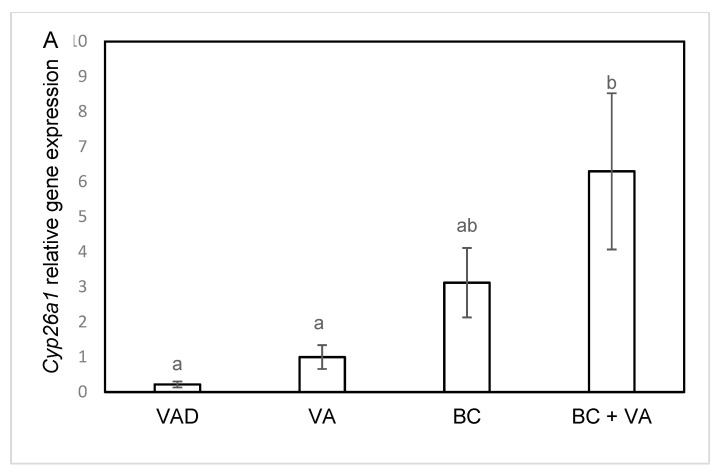

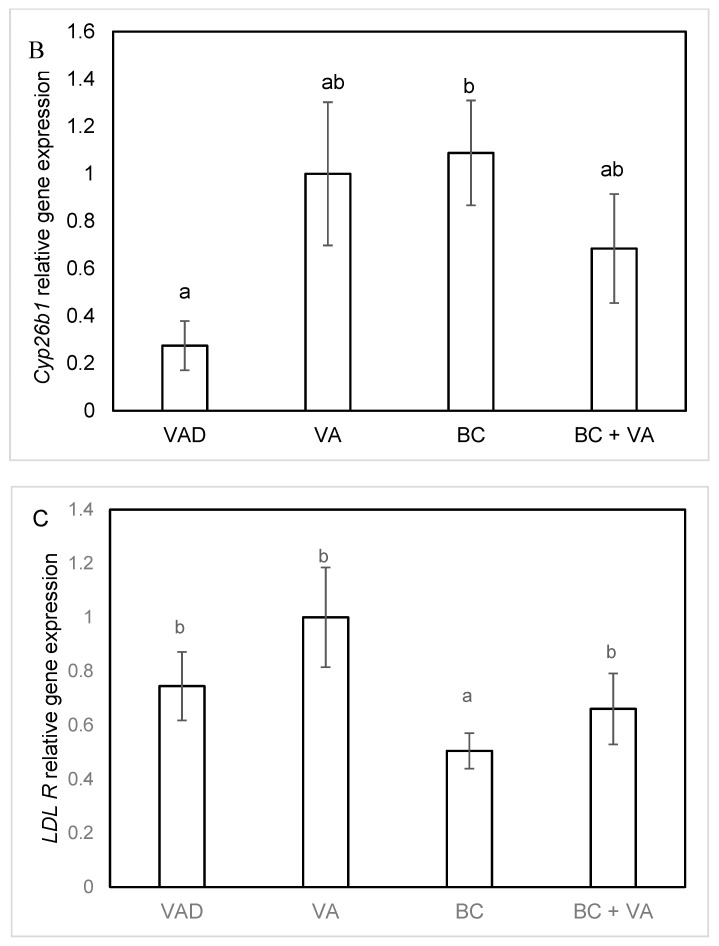
Liver gene expression of *CYP26A1* (**A**) *CYP26B1* (**B**) and LDL R (**C**) in apoE^−/−^ mice, fed with VAD diet, VA, BC or BC+VA. Liver mRNA levels of the indicated genes were detected by real-time PCR. GAPDH was used as a reference gene. Data are means ±SE, n = 5–8 in each group. Mean values (a,b) not sharing a common superscript were significantly different by one-way ANOVA and Tukey post-hoc test comparison (*p* < 0.05). VAD = Vitamin A deficient, VA = vitamin A, BC = β-carotene.

Hepatic mRNA levels of the RA catalyzing enzyme, *Cyp26A1*, were higher in the BC+VA group compared to VAD (Figure 5A). The mRNA levels of *Cyp26b1* were elevated in the BC group compared to VAD (Figure 5B). This change can be attributed to higher retinoid levels in the animal tissues of BC and BC+VA groups. Hepatic mRNA expression for the LDL receptor was lower in the BC group compared to the VAD group (Figure 5C). No change was detected in liver and adipose tissue mRNA levels of the following genes involved in BC and VA metabolism: BCO1, BCO2, Raldh1, Rbp4 (data not shown). 

## 4. Discussion

In the current study performed in apoE^−/−^ mice after weaning, we have demonstrated that a natural BC enriched-diet, composed of all-trans and 9-cis BC isomers, inhibited atherogenesis compared to the VAD diet, while VA did not. Dietary BC, as an exclusive source for VA, compensated over the reduced hepatic VA and the absence of brain VA caused by the VAD diet. We also demonstrated that the 9-cis and all-trans BC stereoisomers were both present and distributed differently in the animal tissues. Unexpectedly, we found that only in adipose tissue, BC accumulation was much higher in mice fed BC alone compared to mice fed a similar dose of BC combined with VA.

Our first aim was to study the effect of a VA-containing vs. VAD diet. Liver is the main organ of VA accumulation [42], and as expected, hepatic VA levels were significantly higher in VA supplemented mice compared to VAD mice. Additional indication for the hepatic VA levels is the reduced mRNA levels of *Cyp26a1* and *Cyp26b1*. These enzymes degrade retinoic acid, the most active metabolite of VA [43]. As expected, *Cyp26a1* levels in BC+VA were significantly higher compared to VAD, and *Cyp26b1* mRNA levels were elevated in the BC compared to the VAD group. The reduction in hepatic VA in the VAD diet was similar to our observation in older, middle age apoE^−/−^ mice [28], and in other mouse strains: Aryl hydrocarbon receptor (AHR)-null mice [44] and BALB/c mice [45]. While adipose tissue and renal VA levels were not affected by VAD diet, in the brain this diet diminished VA to undetectable levels. Our findings suggest that VA levels in the brain tissue are more sensitive to a VAD diet compared with other peripheral tissues. The mechanism of the differential effect of VAD on the brain is unknown. Yet, as VA is essential for the development of the brain and for behavioral and cognitive functions [46], this finding highlights the importance of avoiding the effects of VAD in many organs including the brain.

Our second aim was to study whether dietary enrichment with BC is capable of replenishing the VA levels. In mice treated only with BC, hepatic, adipose tissue, renal and brain VA levels were restored and were comparable to mice treated with VA. These results are in accord with our previous study in middle age apoE^−/−^ mice [28] and with human studies performed in vegetarian children, showing that serum VA status is similar to omnivores [47,48]. 

Our third aim was to study the effect of the combination of BC and VA on their levels in tissues. In the liver, BC+VA treatment resulted in a two-fold elevation of VA compared to mice fed BC or VA alone. However, renal and brain VA levels in mice fed BC+VA were not elevated compared to BC or VA alone. Interestingly, adipose tissue BC levels in adipose tissue of mice fed BC+VA were much lower compared to mice fed BC alone. It has been suggested that both liver and adipose tissue are the main storage organs for VA and BC [49]. In our study, adipose tissue BC concentration in mice fed BC alone were about 30% of the hepatic levels, while in mice fed the combination diet of BC+VA, BC levels were only 4.5% of the hepatic stores. The physiological explanation for the lower accumulation of BC in the adipose tissue in the combined treatment of VA+ BC is not clear. Studies in cattle have shown that high dietary VA decreased both the plasma and hepatic content of carotenoids, with no effect on adipose tissue carotenoids [30]. Wassef et al. found that BC injection to pregnant mice, receiving excess dietary VA, resulted in undetectable BC levels in the embryos, whereas in the embryos of mothers fed a lower dose of VA, absorption and accumulation of BC was unharmed [50]. These results imply that dietary VA has the potential to regulate the bio-distribution of BC. 

The fourth aim was to study whether 9-cis and all-trans BC stereoisomers are differently distributed in tissues. As expected, only low levels of both BC isomers were found in the tissues of the BC-unfortified, VAD and VA mice. Fortification of the diet with BC from the alga *Dunaliella bardawil* led to an elevation of 9-cis BC levels and to a higher ratio of 9-cis to all trans. Interestingly, while in plasma 9-cis levels were 12.6% of total BC in BC group and 10% in BC+VA group; in the liver, the levels of 9-cis were much higher and comprised 45.5% of the hepatic BC in mice fed BC+VA. The precise biological role of the different carotenoid isomers is unclear, yet 9-cis BC may affect atherogenesis through its conversion to 9-cis RA, a ligand of the nuclear receptor RXR, which is known to regulate metabolic pathways inhibiting atherogenesis [51,52]. Our previous study has demonstrated that high 9-cis BC *Dunaliella bardawil*, unlike synthetic all-trans BC, actually protects against atherogenesis in LDL receptor knockout mice [25]. 

The last aim of the study was to investigate whether BC affects atherogenesis in mice fed a VAD diet from weaning to adulthood. We found that in these mice, BC inhibited atherogenesis compared to VAD, similar to our previous study, showing that in middle age apoE^−/−^ mice, a diet enriched with BC inhibits atherogenesis accelerated by VAD [28]. Moreover, VA supplementation failed to inhibit atherogenesis. Several studies established a negative association between consumption of green leafy and yellow-orange vegetables, containing high carotenoids levels, atherosclerosis and cardiovascular disease [53,54]. In humans, carotenoids levels were inversely associated with markers of inflammation [55] and dietary BC was inversely associated with the risk for myocardial infarction [56]. In a recent study, we showed that serum carotenoids correlated inversely with several parameters of body fat composition, glucose regulation, and cardio-metabolic risk factors in humans. However, retinol did not correlate with any of these parameters [57]. 

We have used a remarkably high dose of BC in our mouse models since the development and progression of atherosclerosis is extremely fast compared to atherogenesis in human. It is also known that BC concentration in human plasma is much higher than its level in mice, and therefore extremely high dietary BC is required to reach detectable amounts of BC in the mouse plasma. Regarding human relevance, BC can be provided as a food supplement, and indeed, our human trials have shown that 60 mg BC per day, supplemented as *Dunaliella bardawil* capsules, is sufficient to increase plasma BC significantly [58,59,60]. 

Data regarding VA, VAD and their effect on cardiovascular risk factors and cardiovascular outcome are inconsistent [8,61,62,63]. Therefore, it is of great importance to further investigate whether natural BC supplementation is beneficial over VA supplementation regarding their effect on the atherogenic process and cardiovascular outcome. The results of the current study suggest that dietary fortification with natural algal extract of BC may be beneficial for improving VA status. 

## Supplementary Materials

The following are available online at https://www.mdpi.com/2072-6643/12/6/1625/s1, Table S1: Tissue distribution of VA and total BC.
