# Supplementary material for: Dietary β-Carotene Rescues Vitamin A Deficiency and Inhibits Atherogenesis in Apolipoprotein E-Deficient Mice
Source: Nutrients. 2020 Jun 1;12(6):1625. doi: 10.3390/nu12061625 (PMC7352614; doi:10.3390/nu12061625)
Supplement: Supplementary file 1 [file nutrients-12-01625-s001.pdf]

Supplementary Table 1. Tissue distribution of VA and total BC.

	Experimental Groups			
**Parameters**	**VAD**	**VA**	**BC**	**BC+VA**
Plasma total BC ng/ml	0.032±0.007[a]	0.029±0.010[a]	0.126±0.019[b]	0.100±0.02[ab]
Percent plasma 9-cis BC of total BC	22.3±1.0	21.8±1.7	24.1 ± 1.0	25.9.0±1.0
Liver total BC ng/g	17.6±6.1[a]	27.0±17.1[a]	1,018.0 ± 120.0[b]	1,162 .0±335.0[b]
Percent liver 9-cis BC of total BC	21.6±8.3	ND	40.7±7.7	45.5±3.1
Liver VA ug/g	15.0 ±2.2[a]	110.1±13.1[b]	109.4±25.6[b]	233±30·0[c]
Adipose tissue total BC ng/g	75±19.5[a]	106±25[a]	384±113[b]	51±10.3[a]
Adipose tissue 9-cis BC of total BC	34.0±6.4	29.0±5.6	38.8±1.7	18.0±0.6*
Adipose tissue VA ng/g	62.0±9.2[a]	89.2±14.2[a]	161.1±28.1[ab]	250.0±69.2[b]
Brain total BC ng/g	9.6±4.5[a]	10.2±3.0[a]	20.0± 8.2[a]	18.6 ±2.6[a]
Percent brain 9-cis BC of total BC	ND	11.3±3.9	17.9±3.7	17.3±2.9
Brain VA ng/g	ND[a]	3.5±1.2[b]	4.0±1[b]	4.1±1[b]
Renal total BC ng/g	38.0±14.9[ab]	20.3±3.1[a]	256.1±59[c]	157.5±22[bc]
Percent Renal 9-cis BC of total BC	11.7±4.2	22.7±5.8	37.7±2.2	29.6±2.4*
Renal VA ng/g	29.0±6.5[a]	58.6±11.3[a]	51.1±8.4[a]	51.4±10.7[a]

BC, percent BC of total BC and VA in plasma and tissues. Mean values within a row (a–c) not sharing a common superscript were significantly different by one way ANOVA and Tukey post hoc or Kruskal Wellis test comparison $p<0.05$). t-test was used to test the difference between 9-cis BC percent in BC group compared to BC+A group ($p<0.05$). * significantly different by t-test (BC vs BC + Vit A, $p<0.05$), * Total BC is the sum of 9-cis and all-trans BC. VAD = Vitamin A deficient , VA = vitamin A, BC= β-carotene, ND=not detected.
